# Dexmedetomidine Inhibits Gasdermin D-Induced Pyroptosis *via* the PI3K/AKT/GSK3β Pathway to Attenuate Neuroinflammation in Early Brain Injury After Subarachnoid Hemorrhage in Rats

**DOI:** 10.3389/fncel.2022.899484

**Published:** 2022-06-21

**Authors:** Boyang Wei, Wenchao Liu, Lei Jin, Shenquan Guo, Haiyan Fan, Fa Jin, Chengcong Wei, Dazhao Fang, Xin Zhang, Shixing Su, Chuanzhi Duan, Xifeng Li

**Affiliations:** Neurosurgery Center, Department of Cerebrovascular Surgery, The National Key Clinical Specialty, The Engineering Technology Research Center of Education Ministry of China on Diagnosis and Treatment of Cerebrovascular Disease, Guangdong Provincial Key Laboratory on Brain Function Repair and Regeneration, The Neurosurgery Institute of Guangdong Province, Zhujiang Hospital, Southern Medical University, Guangzhou, China

**Keywords:** Dex, subarachnoid hemorrhage, pyroptosis, early brain injury, microglia

## Abstract

Subarachnoid hemorrhage (SAH) is one kind of life-threatening stroke, which leads to severe brain damage. Pyroptosis plays a critical role in early brain injury (EBI) after SAH. Previous reports suggest that SAH-induced brain edema, cell apoptosis, and neuronal injury could be suppressed by dexmedetomidine (Dex). In this study, we used a rat model of SAH to investigate the effect of Dex on pyroptosis in EBI after SAH and to determine the mechanisms involved. Pyroptosis was found in microglia in EBI after SAH. Dex significantly alleviated microglia pyroptosis *via* reducing pyroptosis executioner GSDMD and inhibited the release of proinflammatory cytokines induced by SAH. Furthermore, the reduction of GSDMD by Dex was abolished by the PI3K inhibitor LY294002. In conclusion, our data demonstrated that Dex reduces microglia pyroptosis in EBI after SAH *via* the activation of the PI3K/AKT/GSK3β pathway.

## Introduction

Subarachnoid hemorrhage (SAH) is a type of hemorrhagic stroke and is defined as bleeding into the subarachnoid space, and which has high morbidity and mortality (van Gijn et al., 2007; Long et al., 2017). SAHs are commonly the result of intracranial aneurysm ruptures, and no effective therapy exists to treat brain injury after SAH (de Oliveira Manoel et al., 2016; Zhang et al., 2018). Currently, the main effect of SAH includes two stages, early brain injury (EBI) and delayed cerebral ischemia (Chen L. et al., 2014; Macdonald, 2014). Recent evidence implied that EBI is a key factor in injury expansion and behavioral deficits (Keyrouz and Diringer, 2007). Mechanisms associated with brain injury due to SAH are still poorly understood, which has resulted in relatively few effective pharmaceutical treatment options. To date, only nimodipine has been proven effective in the treatment of SAH and has been widely used in the clinical applications (Lucke-Wold et al., 2016). Several studies have indicated that cerebral edema, blood–brain barrier (BBB) disruption, oxidative stress, and apoptosis are possible the mechanisms of EBI (Pan et al., 2020). More recently, a study has demonstrated that gasdermin D (GSDMD)-induced pyroptosis may play a critical role in EBI after SAH (Yuan et al., 2020).

Gasdermin D, a member of the gasdermin protein family, has been identified to induce pyroptosis *via* caspase-1 activated inflammasomes in mammals. Specifically, activated caspase-1 cleaves GSDMD at the N-terminal domain to generate GSDMD-N and at the C-terminal domain to generate GSDMD-C. GSDMD-N translocates and binds to the cell inner membrane, forming a non-selective pore, resulting in membrane rupture, cell swelling, and release of interleukin (IL)-1β and IL-18, a process known as pyroptosis (Shi et al., 2015). Accumulating evidence has demonstrated that this GSDMD-induced pyroptosis may play a critical role in some diseases associated with neuroinflammation, including, SAH, Alzheimer's disease, and cerebral ischemia (Voet et al., 2019). Therefore, reducing pyroptosis could be a potential therapeutic target against neuroinflammation.

Dexmedetomidine (Dex), a highly selective α2 adrenergic receptor agonist with sedative, analgesic, and opioid-attenuating effects, is widely used in the intensive care unit for critically ill patients. A previous study showed that the administration of Dex in critically ill patients could improve cardiovascular and ventilatory outcomes during the intensive care unit stay (Castillo et al., 2019). Several other studies have shown that Dex exerts protective effects against apoptosis, necrosis, and autophagy in the brain both *in vitro* and *in vivo* (Cui et al., 2015; Kong et al., 2017; Liu et al., 2020). Dex has been shown to attenuate early brain injury in rats with SAH by suppressing the activation of the TLR4/NF-κB pathway (Yin et al., 2018). In addition, Dex-mediated protection against brain injury induced by sepsis has been demonstrated, which is thought to be mediated *via* inhibition of astrocyte pyroptosis (Sun et al., 2019). However, whether Dex can inhibit pyroptosis in early brain injury (EBI) following SAH remains unknown.

The phosphatidylinositol 3-kinase/protein kinase 3 (PI3K/AKT) signaling pathway has been reported to mediate neuronal proliferation, development, and apoptosis (Arboleda et al., 2010). Glycogen synthase kinase-3 (GSK3β) is a downstream substrate and effector of PI3K/AKT (Sharma et al., 2002). GSK3β is involved in fundamental cellular processes related to regulating energy metabolism, cell growth, and apoptosis (Jacobs et al., 2012). Previously, studies have demonstrated that Dex attenuates apoptosis in the hippocampus and myocardia *via* the activation of the PI3K/AKT/GSK3β pathway (Lv et al., 2017; Cheng et al., 2018). Sophia et al. found that activation of apoptosis by nimbolide, *via* modulation of the PI3K/AKT/GSK3β pathway, caused inhibition of cytoprotective autophagy in oral cancer (Sophia et al., 2018). However, the relation between the PI3K/AKT/GSK3β pathway and Dex in reducing pyroptosis remains unknown. Herein, we aimed to address the possibility of Dex downregulated GSDMD-induced pyroptosis in EBI after SAH by activating the PI3K/AKT/GSK3β pathway in a rat endovascular puncture model of SAH. The rat endovascular perforation model is considered the closest replica of the human condition. Since its development, this model has been extensively used to study early brain injury after SAH (Sehba, 2015).

## Materials and Methods

### Animals

Adult male Sprague-Dawley rats (280–320 g) were obtained from the Animal Experiment Center of Southern (Guangzhou, China). All experimental procedures and animal care were approved by the Southern Medical University Ethics Committee and were by the guidelines of the National Institute of Health. All animals resided in controlled temperature and humidity conditions (22 ± 1°C; 40–60%) with a 12:12-h dark/ light cycle (lights on at 07:00 h; off at 21:00 h). Food and water were available *ad libitum* and adapted to the environment 1 week before the experiments.

### Experimental Design and Groups

In the first experiment, 112 male rats were randomly divided into eight groups (14 rats per group): sham and 3, 6, 12, 24, 48, 72, and 96 h after SAH. The protein expression level and time course of GSDMD, GSDMD-N, pro-caspase-1, and caspase-1 p20 were measured by western blot. The mRNA expression level of GSDMD was measured by qPCR. In an additional study, western blot was also employed to detect whether there was a statistical difference in GSDMD, GSDMD-N, pro-caspase-1, and caspase-1 p20 protein levels among the sham groups at 3,6, 12, 24, 48, 72, and 96 h. Expression distribution was detected by double immunostaining to determine GSDMD and pro-caspase-1 expression in different cell types of the brain in the 24 h after the SAH group. Transmission electron microscopy was conducted in the sham group and 24 h after the SAH group.

In the second experiment, to detect an optimal dose of Dex on pyroptosis after SAH, 84 rats were randomly divided into six groups (14 rats per group): sham, SAH, SAH + vehicle-1, SAH + Dex (15 μg/kg), SAH + Dex (25 μg/kg), and SAH + Dex (45 μg/kg), to determine whether GSDMD-induced pyroptosis could be reduced by Dex at an optimal dose. All the rats were euthanized 24 h after SAH according to the results of the first experiment. The changes in GSDMD and GSDMD-N after SAH caused by different doses of Dex were analyzed by western blot. Neurological scores and brain water content analysis were conducted.

In the third experiment,120 rats were used to explore whether the PI3K/AKT/GSK3β pathway could be activated *via* DEX at 24 h after SAH. Rats were randomly divided into four groups (30 rats per group) sham, SAH, SAH + vehicle-1 (normal saline), and SAH + Dex (optimal dose). Animals were sacrificed for brain tissue 24 h after SAH. The samples were collected for qPCR, ELISA, and fluoro-Jade C (FJC) analysis. We used western blot to prove that Dex can activate the PI3K/AKT/GSK3β pathway after SAH.

In the fourth experiment, 150 rats were used to detect whether inhibition of the PI3K/AKT/GSK3β signaling pathway could influence Dex-mediated anti-pyroptosis effects at 24 h after SAH. Rats were randomly divided into five groups (30 rats per group): sham, SAH, SAH + Dex (optimal dose), SAH + Dex (optimal dose) + vehicle-2 [dimethylsulfoxide (DMSO)], and SAH + Dex (optimal dose) + LY294002 (PI3K inhibitor). LY294002 is delivered by intraventricular injection. Neurological scores, qPCR, fluoro-Jade C (FJC)/immunohistochemistry (IHC) analysis, ELISA, and western blotting were performed 24 h after SAH induction. The total number and mortality of rats in each group are shown in Supplementary Table 1.

### Experimental SAH Model

**Figure 1:**
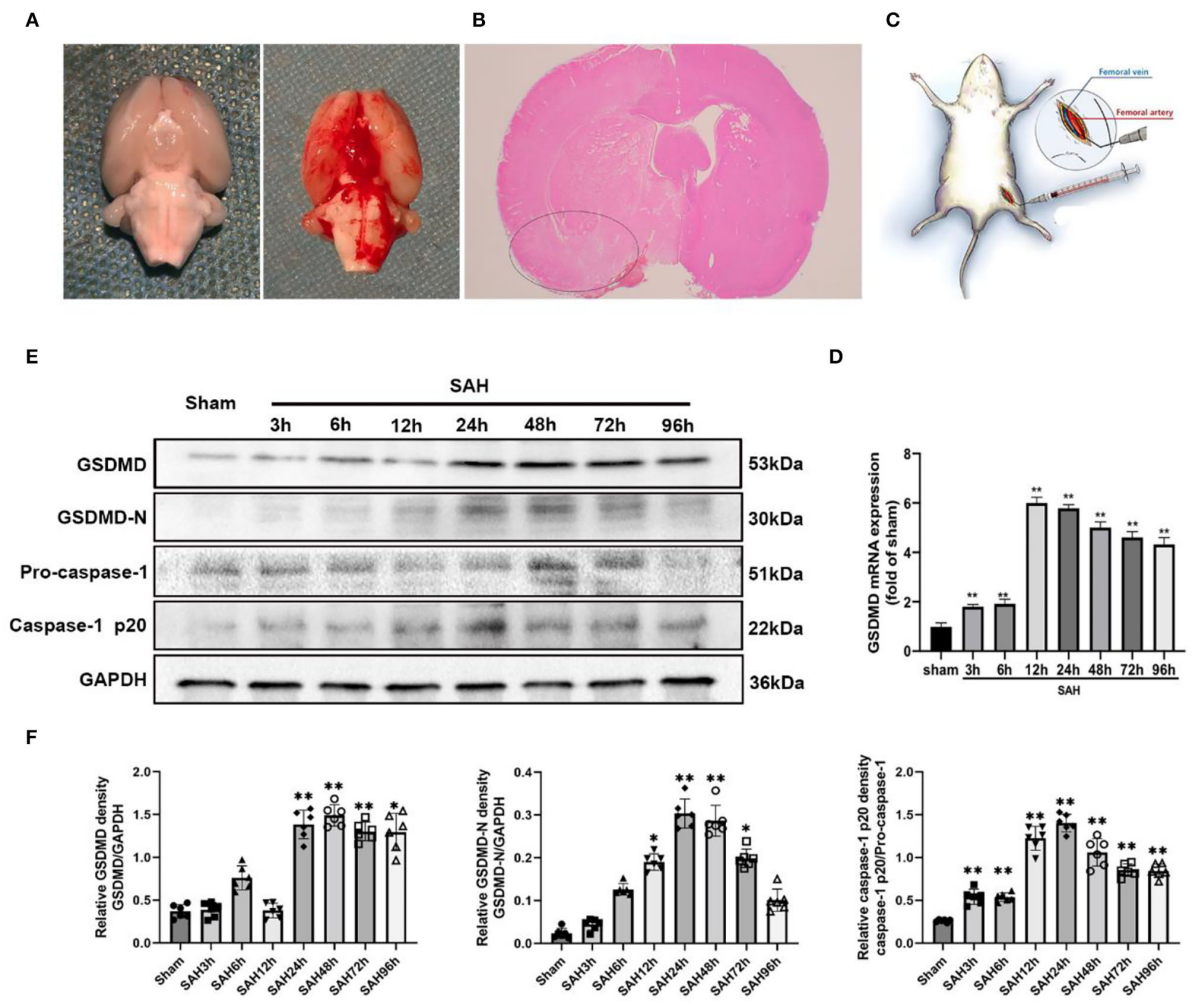
**(A)** Representative image of subarachnoid hemorrhage (SAH) model. **(B)** A schematic indicating the brain region for immunochemistry staining, qRT-PCR, IF staining, and western blotting (black circle). **(C)** Representative image of dexmedetomidine delivery. **(D)** Quantification of GSDMD mRNA level in rat temporal cortex. **(E)** Western blot analysis showed the level of GSDMD, GSDMD-N, pro-caspase-1, and Casepase-1 p20 protein abundance at 3, 6, 12, 24, 48, 72, and 96 h after SAH. **(F)** Quantification of GSDMD, GSDMD-N, and casepase-1 p20 protein level as shown in **(E)**. All values are presented as means ± SD, *n* = 6 in each time point per group. *p < 0.05, **p < 0.01.

The procedure for the SAH model in rats has been described in previous publications with some small modifications (Li R. et al., 2018). Briefly, rats were deeply anesthetized by 1% pentobarbital sodium (40 mg/ kg, i.p.) with a core temperature of 37.5°C maintained throughout the procedure. A sharpened 4-0 nylon suture was inserted rostrally into the internal carotid artery (ICA) to perforate the intracranial bifurcation of the ICA and middle cerebral arteries until resistance was felt. After SAH, the suture was immediately withdrawn to allow blood reperfusion in the ICA. Sham animals underwent the same procedures without vessel perforation. Representative images of brains from Sprague-Dawley rats in sham and SAH groups are shown in Figure 1A. We specifically focused on the basal cortex of the hemisphere (Figure 1B). After the perfusion, rats' brain was placed under a microscope, and the temporal floor cortex was cut out with a microscopic instrument for ELISA, WB, and qPCR research.

### BV-2 Microglia Culture and Treatments

BV-2 microglia cell line was purchased from Jennio Biotech (Guangzhou, China). Cells were cultured in DMEM supplemented with 10% FBS. To mimic the SAH model *in vitro*, BV-2 cells were treated with oxyhemoglobin (OxyHb) at the concentration of 25 μm after 24 h as the previous study described (Lu et al., 2018). *In vitro* Dex treatment assay, cultures were treated with 1 μm Dex (Sigma-Aldrich) 30 min before OxyHb treatment. *In vitro*, PI3K inhibition experiments were conducted by pre-incubating the OxyHb-primed cells with 10 μm LY294002 for 2 h. The doses of Dex and LY294002 were determined according to the previous studies (Sun et al., 2019; Ji et al., 2020).

### Intracerebroventricular Injection

Injection of LY294002 (PI3K inhibitor) into the lateral ventricle was performed as previously described (Xie et al., 2018). LY294002 (Sigma-Aldrich, St. Louis, MO, USA) was dissolved in DMSO at 1 mg/ml. LY294002 and vehicle-2 (DMSO) were administered slowly at 1 μl/min into the left lateral ventricle after 30 min of SAH using a 10-μl Hamilton microsyringe (microliter no. 701; Hamilton Company, Switzerland) under the guidance of a stereotaxic instrument (Stoelting Company, USA) under anesthesia. The microsyringe was kept in the left lateral ventricle for an additional 5 min after administration and then withdrawn slowly. Finally, the skull hole was filled with bone wax and the incision was sutured carefully.

### Neurological Score Evaluation and SAH Grade

Neurological scores were evaluated by an independent investigator blinded to the procedural information using a previously described modified Garcia scoring system (Zeng et al., 2017). In the Garcia test, spontaneous activity, spontaneous movement of all limbs, forelimbs outstretching, climbing, a touch of the trunk, and vibrissae touch were assessed. These tests were scored from 0 to 3 or 1 to 3, and the total score ranged from 3 to 18 (Supplementary Table 2). Higher scores represented better neurological function. The SAH grade evaluation included six areas of the basal surface of the brain score from 0 to 3 based on the amount of subarachnoid blood, Grade 0: no subarachnoid blood, 1: minimal subarachnoid blood, 2: moderate blood clot with recognizable arteries, and 3: blood clot obliterating all arteries within the segment (Sugawara et al., 2008). SAH grade evaluation also was evaluated by an observer who was blinded to the treatment conditions. In this study, we eliminated the mild SAH rats (total scores <8) as the SAH grade was not significantly different for neurological impairments among the sham and treated and untreated mild SAH groups.

### Brain Water Content Analysis

At 24 h after SAH, we removed the brains and divided each quickly into four parts: the left hemisphere, right hemisphere, cerebellum, and brainstem. Each part was weighed immediately to obtain the wet weight (WW) and dried in an oven for 24 h at 105°C to obtain the dry weight (DW). The percentage of water content was calculated as follows: [(WW-DW)/WW] × 100%.

### Evaluation of BBB Permeability

Quantitative analysis of BBB permeability was evaluated *via* EB dye as previously described (Liu et al., 2019). Briefly, the rats were anesthetized and administrated intravenously 2% EB (4 ml/kg was injected intravenously after at 24 h SAH) intravenously *via* the femoral vein and circulated for 2 h. Under deep anesthesia, the brains were removed and the brain samples were immediately separated into the left and right hemisphere. Subsequently, the hemispheres were incubated in 50% trichloroacetic acid solution (2 ml) and centrifuged at 15,000 × g for 30 min. Then, the supernatant (1 ml) was diluted with ethanol (1:3). Finally, the resultant supernatant was measured at an excitation wavelength of 620 nm and an emission wavelength of 680 nm with an automatic microplate reader. The data were quantified using GraphPad Prism 8 (GraphPad Software, Inc., San Diego, CA).

### Dex Administration

Dexmedetomidine was delivered intravenously to rats within 30 min after induction of SAH. Dex (0.5 mg; Sigma-Aldrich) was dissolved in 50 ml of normal saline. The femoral vein was dissected and exposed with blunt dissection, and Dex was slowly injected for 30 s using a 1-ml syringe with a curved needle (Figure 1C). Then, the needle was removed, the femoral vein was made hemostatic, and the incision was closed carefully. In the second experiment, the three different doses of Dex were 15, 25, and 35 μg/kg. The final injection volume was determined by the weight of each rat. Rats in the sham group were infused with an equal amount of normal saline without Dex.

### Hematoxylin and Eosin Staining

At 24 h after SAH, rats were anesthetized and rapidly perfused with 300 ml of 0.01 M PBS (pH 7), followed by 500 ml 4% paraformaldehyde solution. Then, the brains were removed and postfixed in the same fixative for 48 h at 4 °C. After dehydration and vitrification, tissue samples were embedded in paraffin, and 4-μm sections were prepared (Xie et al., 2015). The sections were stained with hematoxylin for 5 min and eosin for 10 s. After dehydrating in graded ethanol and clearing in xylene, images were obtained using a microscope (Leica-DM2500, Leica Microsystems, Wetzlar, Germany).

### Immunohistochemical Staining

Coronal brain sections (4-μm thickness, paraffin-embedded) were prepared as mentioned above. Antigen retrieval was performed by heat treatment in a microwave oven for 21 min in Tris-ethylene diamine tetraacetic acid buffer solution (0.05 mol/l Tris, 0.001 mol/L EDTA; pH 8.5). Endogenous peroxidase activity was quenched using 3% H_2_O_2_ for 10 min followed by washing with PBS. Sections were then blocked in 5% bovine serum albumin (BSA) for 20 min and were incubated overnight at 4°C with the following primary antibody: rabbit anti-GSDMD (1:400, Abcam, ab219800). After washing with PBS, the slides were incubated with biotinylated goat anti-rabbit IgG for 20 min and then incubated with horseradish peroxidase-streptavidin reagent for 20 min at room temperature. Finally, immunoreactivity was detected using 3,3-diaminobenzidine. Images were captured on a light microscope (Leica-DM2500, Leica Microsystems).

### Fluoro-Jade C Staining

Fluoro-Jade C (FJC) staining was performed to detect degenerated neurons as previously reported with some modifications (González et al., 2020). Briefly, rat brain sections were prepared as mentioned above, then immersed with alcohol solution (1% sodium hydroxide in 80% ethanol) and 70% ethanol for dewaxing, and then immersed in 0.06% potassium permanganate solution for 10 min. Subsequently, the sections were transferred into a 0.0001% FJC (AG325, Millipore, Darmstadt, Germany) working solution for 30 min and then washed in xylene and coverslipped with paramount TM mounting medium (Cwbiotech, Guangzhou, China). Images were captured using a fluorescence microscope (Leica-DMI8, Leica Microsystems, Wetzlar, Germany). The FJC-positive cells were counted in six slides per mouse by a blinded observer. Data were expressed as the ratio of FJC-positive cells (relative to the sham group).

### Immunofluorescence Staining

Immunofluorescence (IF) staining was performed as previously described but with some modifications (Chen et al., 2015). Briefly, coronal paraffin-embedded 4-μm thickness brain sections were prepared as mentioned above. The sections underwent antigen retrieval and were then blocked in 5% BSA for 1 h. After blocking, sections were incubated overnight at 4°C with the following primary antibodies used: rabbit anti-GSDMD (1:200, Ab219800, Abcam), rabbit anti-caspase-1 (1:200, Ab238972, Abcam), rabbit anti-p-AKT (1:200, 4,060, Cell Signaling Technology), rabbit ani-p-GSK3β (1:200, 5,558, Cell Signaling Technology), and mouse anti-Iba1 (1:200; GB12105, Servicebio). The next day, the slices were washed with PBS and incubated with secondary antibodies: donkey anti-goat Alexa 488 (1:500, A11055, Invitrogen) and donkey anti-rabbit Alexa 555 (1:500, A31572, Invitrogen) for 1 h at room temperature. After washing three times with PBS, the sections were re-stained by 4′6-diamidino-2-phenylindole (DAPI) for 10 min. Then, images were acquired using a fluorescence microscope (ZEISS-AXIO Scope. Al, Germany).

### Quantitative Real-Time PCR

Quantitative real-time PCR (qRT-PCR) was performed and analyzed as previously described (Livak and Schmittgen, 2001). Rat temporal floor cortex samples were homogenized in TRIzol reagent (Sigma-Aldrich) according to the manufacturer's instructions. Total RNAs were isolated and reverse-transcribed to cDNA with a prime script RT reagent kit (RR047A, Takara Bio Inc., Shiga, Japan). For qRT-PCR, a 7500 real-time PCR thermocycler (Applied Biosystem) was used. Real-time RT-PCR was performed in a total volume of 10 μl containing 1 μl of cDNA, 0.6 μl of primers, and 8.4 μl of SYBR Green PCR Master Mix (RR820A, Takara Bio Inc.). The program steps were 30 s at 95°C, 40 cycles of 5 s at 95°C, and 30 s at 60°C, followed by melt curve analysis. Gene expression was quantified with standard samples and normalized with β-actin. The primer sequences are listed in Supplementary Table 3.

### Western Blot

Rats were transcardially perfused with cold PBS (0.1 M, pH 7.4), and the ipsilateral cortex was quickly dissected for western blot analysis as described previously (Li et al., 2019). Briefly, protein samples from the left cerebral cortex were isolated and prepared using RIPA lysis buffer (Cwbio, Guangzhou, China), and the total protein concentration was measured with a bicinchoninic acid protein assay kit (Genecopoeia, Rockville, MD, USA). Equal amounts of protein (50 μg) from different brains were separated by sodium dodecyl sulfate-polyacrylamide gel electrophoresis (Cwbio, Guangzhou, China) and transferred to a polyvinylidene difluoride filter membrane. Membranes were then blocked in 5% non-fat milk dissolved in Tris-buffered saline with 0.1% Tween 20 for 1 h, and membranes were incubated with the following primary antibodies: rabbit anti-GSDMD (1:1,000, Ab219800, Abcam), rabbit anti-caspase-1 (1:1,000, Ab238972, Abcam), rabbit anti-p-AKT (1:1,000, 4,060, Cell Signaling Technology), anti-AKT (1:1,000, 4,685, Cell Signaling Technology), rabbit anti-p-GSK3β (1:1,000, 5,558, Cell Signaling Technology), rabbit anti-GSK3β (1:1,000, 12,456, Cell Signaling Technology), rabbit anti-ZO-1 (1:1,000, ab221547, Abcam), anti-claudin-5 (1:1,000, ab236066, Abcam), and rabbit anti-GAPDH (1:1,000, 5,174, Cell Signaling Technology) overnight at 4°C and then washed three times in Tris-buffered saline for 5 min with 0.1% Tween 20. Afterward, the blots were incubated with a secondary antibody, peroxidase-conjugated goat anti-rabbit IgG (1:1,000, 7,074, Cell Signaling Technology), and identified using the ECL western blotting detection system (Millipore, Darmstadt, Germany). The intensities of blots were quantitated with ImageJ software (ImageJ 1.5, National Institutes of Health, Bethesda, MD, USA). GAPDH was used as the internal loading control.

### ELISA

At 24 h after SAH, rats were deeply anesthetized and serum samples from the left hemisphere were obtained and stored at −80°C until use. The concentrations of IL-10, IL-1β, and IL-18 in brain tissue lysates were analyzed using commercial ELISA kits (BMS625, BMS630, and BMS629, Invitrogen) according to the manufacturer's instructions. The final concentration of cytokines was obtained from the determined standard curve of absorbance.

### Transmission Electron Microscopy

We performed transmission electron microscopy (TEM), as previously reported (Xu et al., 2020). Briefly, after euthanasia, the basal cortex of the hemisphere in SAH groups and the corresponding area in the sham group of the rats were collected and processed into 100-nm sections, which were then stained with 2.5% glutaraldehyde and then postfixed in 1% osmium tetroxide. Finally, the sections were visualized using transmission electron microscopy (Hitachi, Japan). Microglial cell bodies can be discerned from those of other cell types by their small size (3–6 μm), electron-dense cytoplasm, and characteristically bean-shaped nuclei (Savage et al., 2018). The typical morphological features of pyroptosis are the intact nucleus and the cell membrane pores, which are also the important signs distinguishing them from apoptosis (Jorgensen and Miao, 2015).

### TUNEL Staining

To detect cell apoptosis, terminal deoxynucleotidyl transferase dUTP nick end labeling (TUNEL) was performed using the One-Step TUNEL Assay Kit (Beyotime, China) at 24 h after SAH. Briefly, brain specimens were immersed in a TUNEL mixture for 2 h at 37.5°C, followed by a stained with DAPI. The data were expressed and analyzed in the same procedure as for FJC staining.

### Statistical Analysis

All data were presented as means ± standard deviation. All statistical analyses were performed using GraphPad Prism 8 (GraphPad Software, Inc., San Diego, CA). After checking for normal distribution, differences between two groups were analyzed with Student's *t*-test (two-tailed), and data were analyzed by one-way analysis of variance (ANOVA) with *post hoc* Tukey's test or Dunnett's test applied to assess multiple comparisons. Nonparametric data were analyzed using the Kruskal–Wallis H analysis followed by a Mann–Whitney U test, and *p* < 0.05 was considered statistically significant.

## Results

### Upregulation of GSDMD MRNA and Pyroptosis After SAH

**Figure 2:**
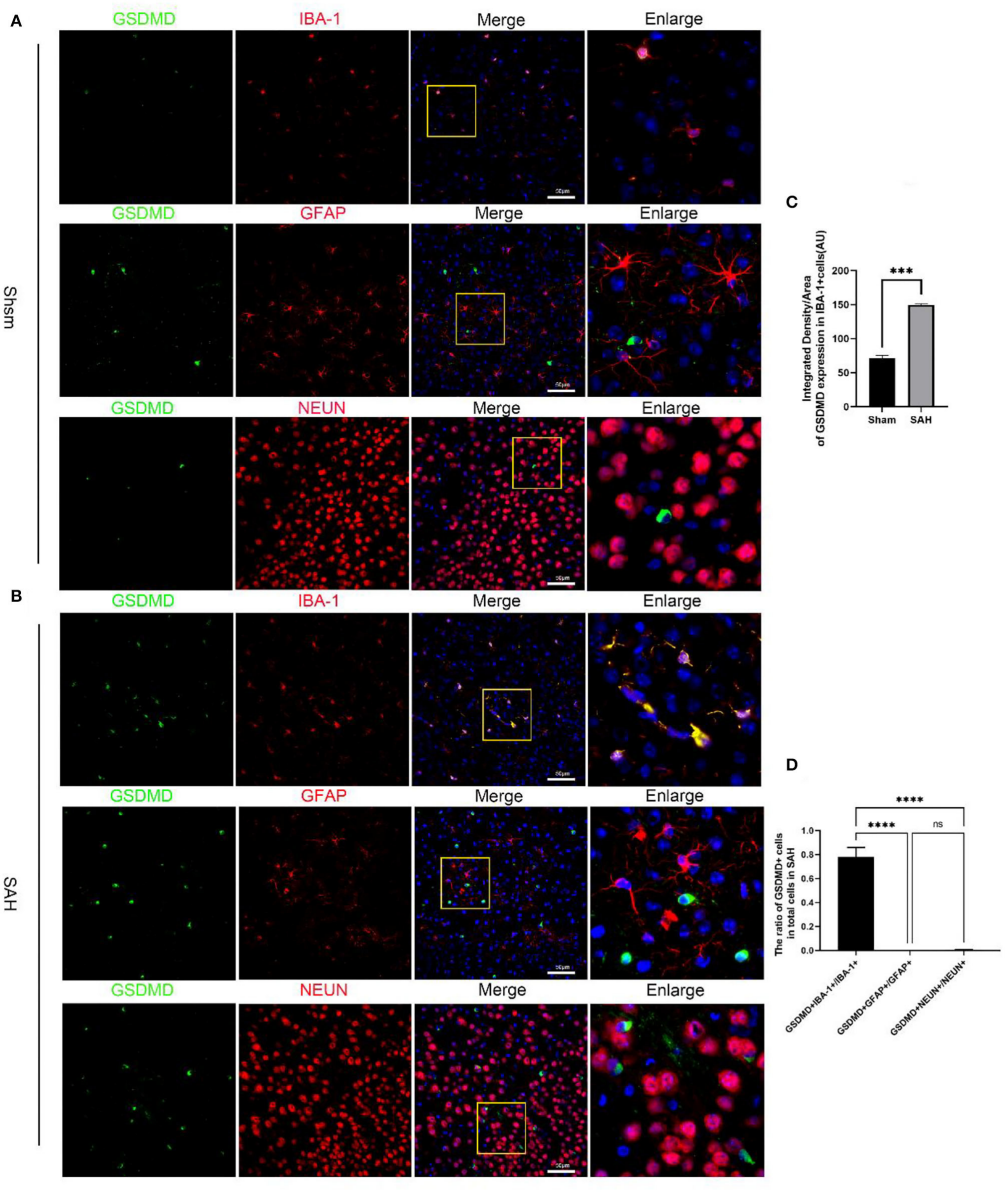
Cellular localization of pyroptosis executioner GSDMD in the ipsilateral hemisphere. **(A)** Representative immunofluorescence staining slices of GSDMD (green) with microglia (IBA1, red), astrocytes (GFAP, red), and neurons (NEUN, red) in the ipsilateral cortex. **(B)** Representative immunofluorescence staining slices of GSDMD (green) with microglia (IBA1, red), astrocytes (GFAP, red), and neurons (NEUN, red) in the ipsilateral cortex at 24 h after SAH. Scale bars = 50 μm. **(C,D)** Quantitative analysis of immunofluorescence staining. All values are presented as means ± SD, *n* = 6 in each group. ***p < 0.001 and ****p < 0.0001.

**Figure 3:**
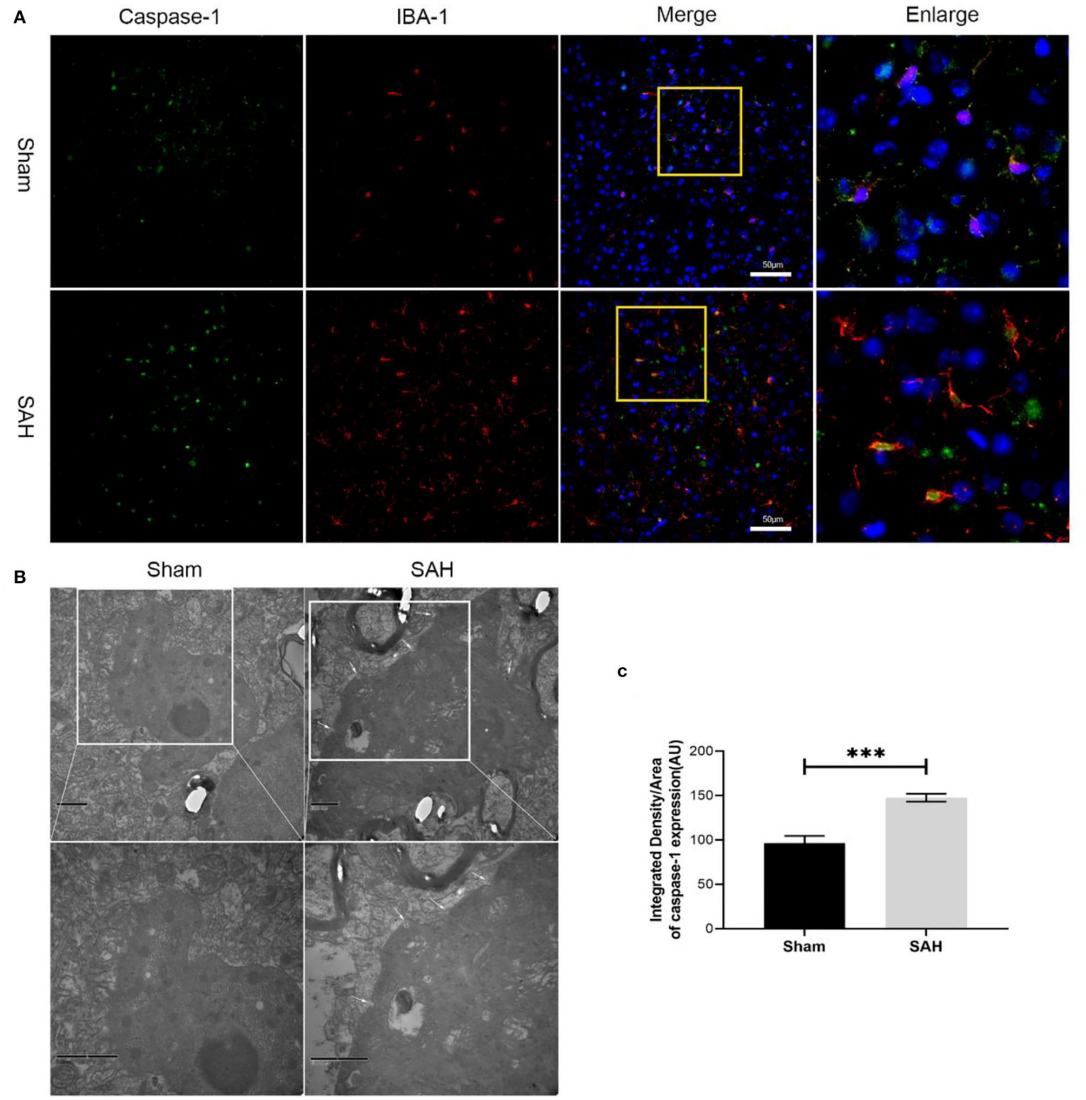
Microglia expresses caspase-1 and representative transmission electron microscopy images. **(A)** Representative images of co-immunofluorescence staining of caspase-1 (green) and microglia (IBA1, red). Scale bars = 50 μm. **(B)** Representative transmission electron microscopy images of microglia in ipsilateral cortex. Write arrow head: membrane pores. Scale bars = 500 nm. **(C)** Quantitative analyses of caspase-1. Bars represent mean ± SD, *n* = 6 in each group. ***p < 0.001.

Quantitative real-time PCR (qRT-PCR) was performed to investigate the mRNA level of GSDMD in the sham and 3, 6, 12, 24, 48, 72, and 96 h groups. The mRNA level of GSDMD was increased after SAH (Figure 1D). WB was performed to investigate the endogenous expression of GSDMD, GSDMD-N, and caspase-1 p20 in the left hemisphere in the sham and 3, 6, 12, 24, 48, 72, and 96 h groups. GSDMD, GSDMD-N, and caspase-1 p20 levels were low in the sham group, but they were elevated immediately after SAH and reached a peak at 24 h after SAH (Figures 1E,F). Double IF staining showed that GSDMD was elevated and expressed in microglia in the cortex of the ipsilateral hemisphere at 24 h post-SAH (Figure 2). Furthermore, microglia also expressed caspase-1 at the same site (Figure 3A). GSDMD and GSDMD-N were the executive factors of pyroptosis and were also important evidence for the identification of pyroptosis. At the same time, using electron microscopy, microglial was found to be pyroptotic in the ipsilateral hemisphere cortex (Figures 3B,C).

### Dex Alleviated EBI After SAH and Reduced the Expression of GSDMD and GSDMD-N at 24 h After SAH

**Figure 4:**
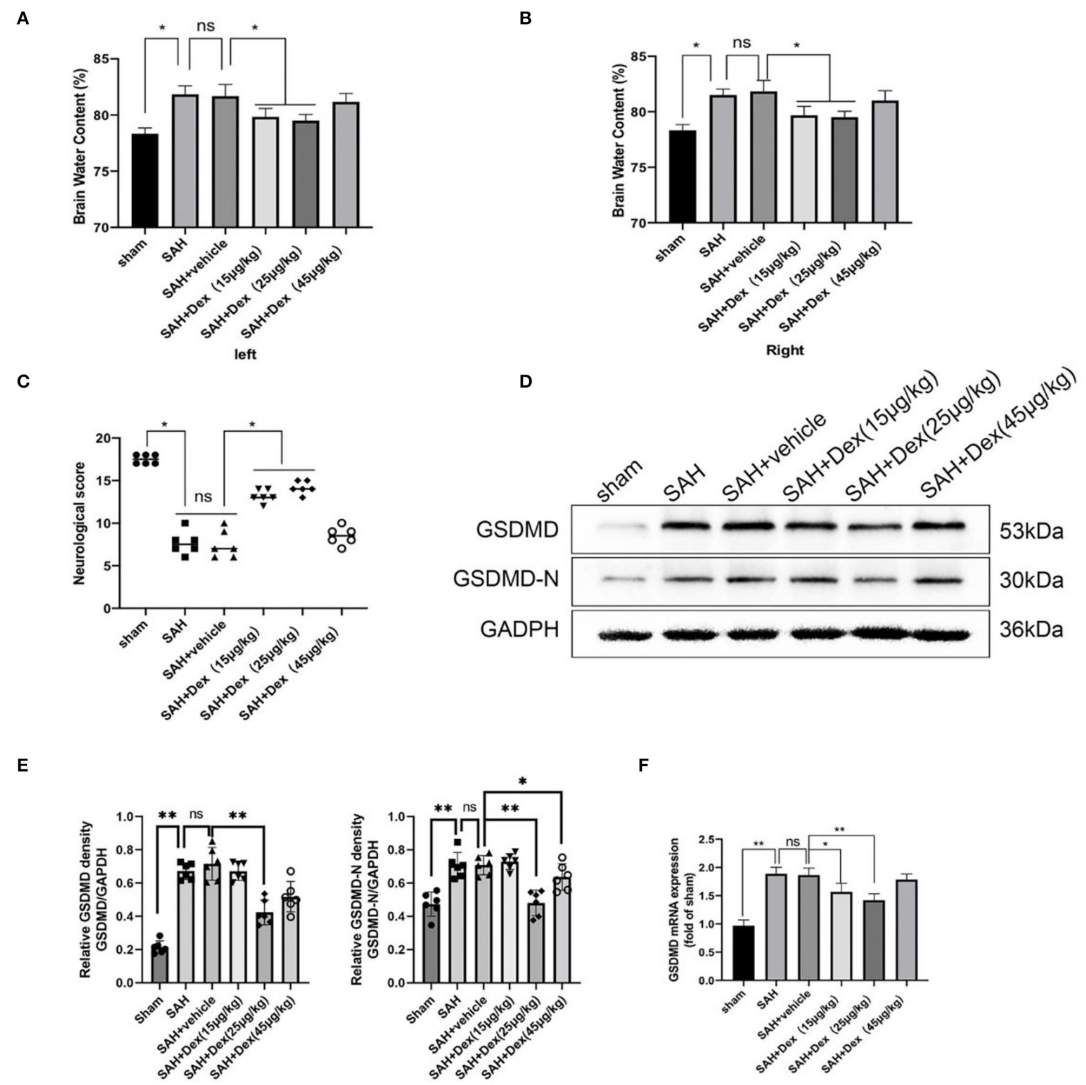
Dex attenuates short-term neurological deficit and brain edema at 24 h after SAH. **(A,B)** Quantification of brain water content 24 h after SAH. **(C)** Neurobehavioral deficits 24 h after SAH. Effect of 25 μg/kg dosage of Dex on the protein levels of GSDMD, GSDMD-N **(D,E)** and GSDMD mRNA expression **(F)**. Values are expressed as the mean ± SD, *n* = 6 in each group. *p < 0.05, **p < 0.01, ns, no significant.

At 24 h after SAH, neurobehavioral activity and brain edema were examined in all groups. A total of three dosages of Dex (15, 25, and 45 μg/kg) were administrated by intravenous injection 30 min after SAH. At 24 h, SAHs caused poorer brain water content and neurological impairment compared with the sham group. No significant differences between the SAH and SAH + vehicle groups in neurological scores and brain edema were observed. At doses of 15 and 25 μg/kg, Dex dramatically ameliorated brain edema and neurological impairment, but no significant differences between the SAH + vehicle and SAH + Dex (45 μg/kg) group were found (Figures 4A–C). However, only administration of Dex at 25 μg/kg significantly reduced GSDMD and GSDMD-N protein expression compared with the SAH + vehicle group (Figures 4D,E). Consistent with the foregoing results, the mRNA level of GSDMD was noticeably decreased after Dex (25 μg/kg) treatment (Figure 4F). These results indicated that Dex, at a dosage of 25 μg/kg, was effective for reducing GSDMD-induced pyroptosis, and therefore, Dex, at a dose of 25 μg/kg, was selected for further studies.

### Dex Treatment Attenuated Neuronal Injury and Alleviated Disruption to the BBB at 24 h After SAH

**Figure 5:**
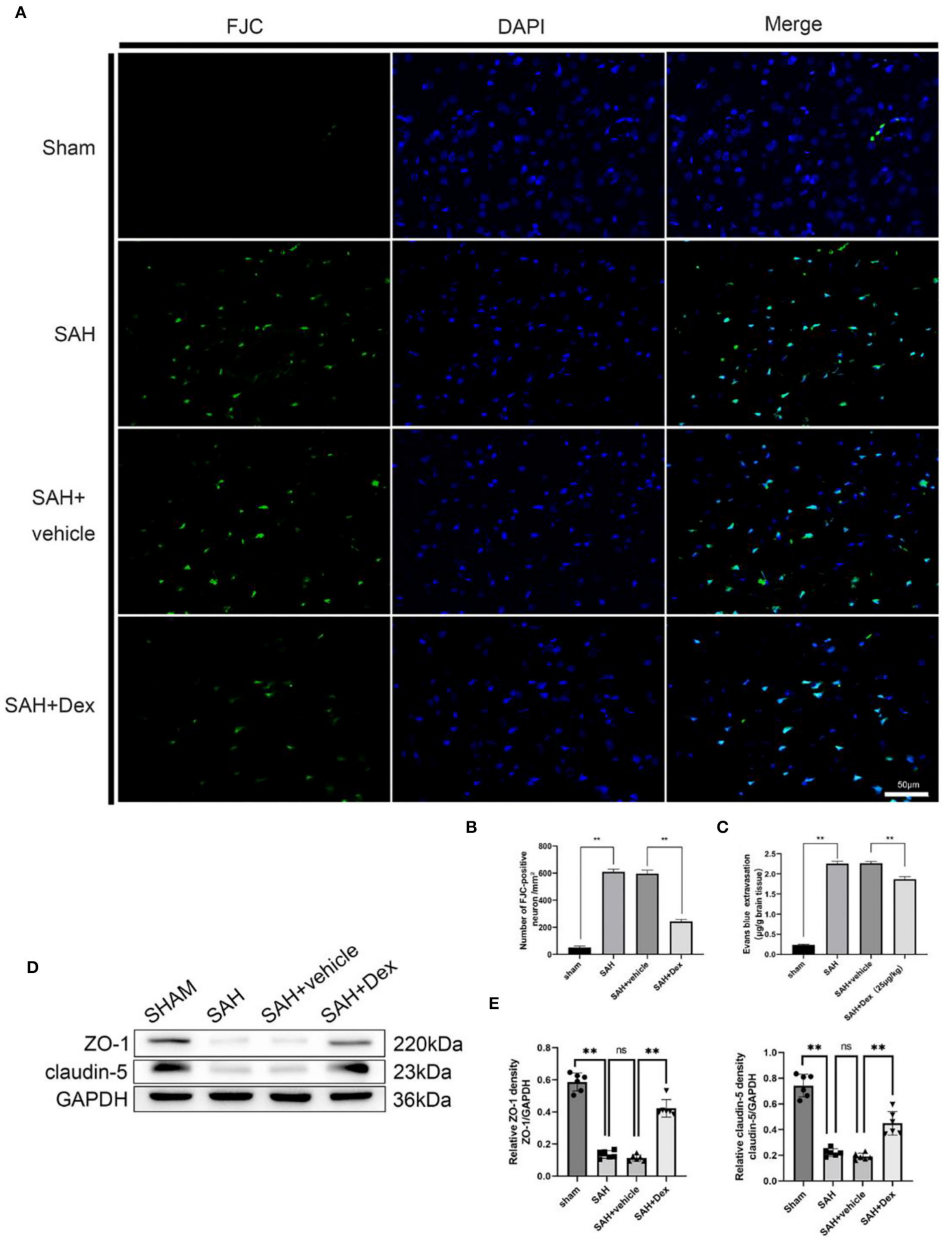
Effects of Dex on neuronal degenerating and BBB disruption at 24 h after SAH. **(A)** Representative microphotographs of Fluoro-Jade C staining (FJC)-positive neurons in the sham, SAH, vehicle, and dexmedetomidine groups at 24 h following operation. **(B)** Quantitative analysis of FJC-positive cells was performed at the ipsilateral cortex. **(C)** Quantitative analysis of Dex administration significantly alleviated the extravasation of EB dyes. **(D)** Representative western blot images. **(E)** Quantitative analysis of western blot. *n* = 6 in each group. Data are expressed as mean ± SD. **p < 0.01. Scale bar = 50 μm.

To further investigate the protective effects of Dex treatment at 24 h after SAH, we estimated the neurodegeneration in the injured cortical region as revealed by FJC staining (Figure 5A). When compared to the SAH + vehicle group at 24 h after SAH, the number of FJC+ cells was significantly decreased in the injured cortical region in Dex-treated SAH rats, whereas no significant difference was found between the SAH and SAH + vehicle groups (Figure 5B). In addition, Dex treatment significantly decreased Evans blue dye extravasation in the left hemisphere compared with the SAH + vehicle group (Figure 5C). WB results showed that Dex could increase the expression of ZO-1 and claudin-5 after SAH (Figures 5D,E).

### Dex Was Involved in the Activation of the PI3K/AKT/GSK3β Pathway, Which Alleviated Pyroptosis in the Cortex at 24 h After SAH

**Figure 6:**
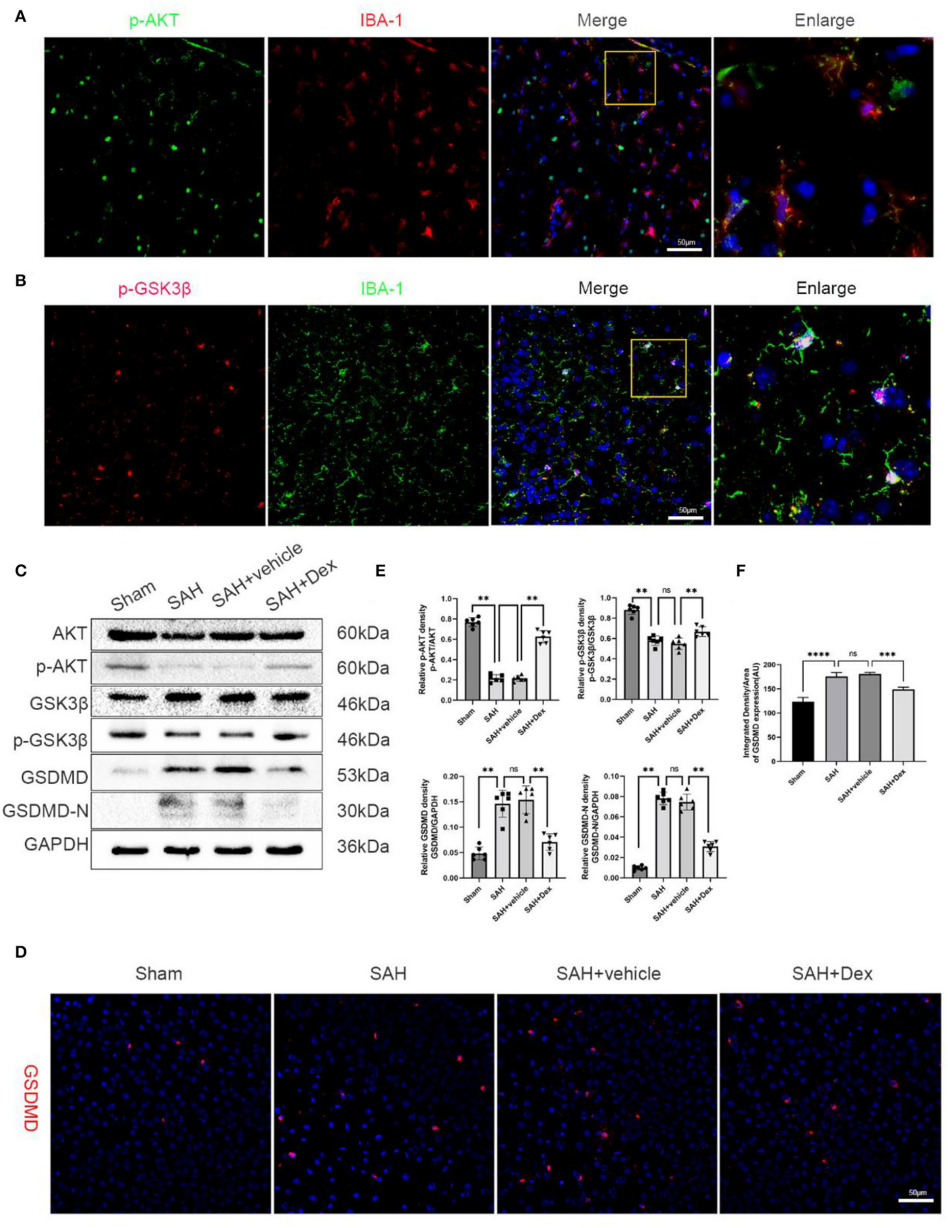
Effects of Dex treatment on PI3K/AKT/GSK3β pathway, protein levels of GSDMD and GSDMD-N. **(A)** Representative images of co-immunofluorescence staining of p-AKT (green) and microglia (IBA1, red) in the ipsilateral cortex at 24 h after SAH. Scale bars = 50 μm. **(B)** Representative images of co-immunofluorescence staining of p-GSK3β (red) and microglia (IBA1, green) in the ipsilateral cortex at 24 h after SAH. Scale bars = 50 μm. **(C)** Representative Western blot images. **(D)** Representative images of immunofluorescence staining GSDMD. Scale bar = 50 μm. **(E,F)** Quantitative analysis of western blot and immunofluorescence staining. *n* = 6 in each group. Data are expressed as mean ± SD. **p < 0.01, ***p < 0.001, ****p < 0.0001.

The PI3K/AKT/GSK3β pathway plays a pivotal role in neuroprotection and anti-apoptotic processes within the central nervous system. As shown in (Figures 6A,B), p-AKT and p-GSK3β were expressed in microglia in the cortex of the ipsilateral hemisphere at 24 h after SAH. To explore the effects of Dex on PI3K/AKT/GSK3β signaling after SAH, we used WB in this study. Dex, at the dose of 25 μg/kg, significantly upregulated the expression of p-AKT and p-GSK3β compared with SAH + vehicle groups (Figures 6C,E,F). Additionally, the expression of GSDMD and GSDMD-N protein was significantly decreased following Dex treatment. The IF of the GSDMD result was consistent with WB results (Figure 6D).

To further confirm our results *in vivo*, the effects of Dex on the PI3K/AKT/GSK3β pathway and pyroptosis were also examined *in vitro*. Consistent with the results *in vivo*, Dex administration significantly increased the protein of p-AKT and p-GSK3β in OxyHb-treated microglia (Supplementary Figures 1A,C). The expression of IBA-1 and GSDMD co-localization was evident in cultured microglia after exposure to OxyHb. In addition, Dex significantly reduced the expression of GSDMD relative to the OxyHb-treated microglia (Supplementary Figures 1B,D).

### Dex Treatment Also Alleviated Apoptosis and Reduced Inflammatory Cytokine Expression in the Cortex at 24 h After SAH

**Figure 7:**
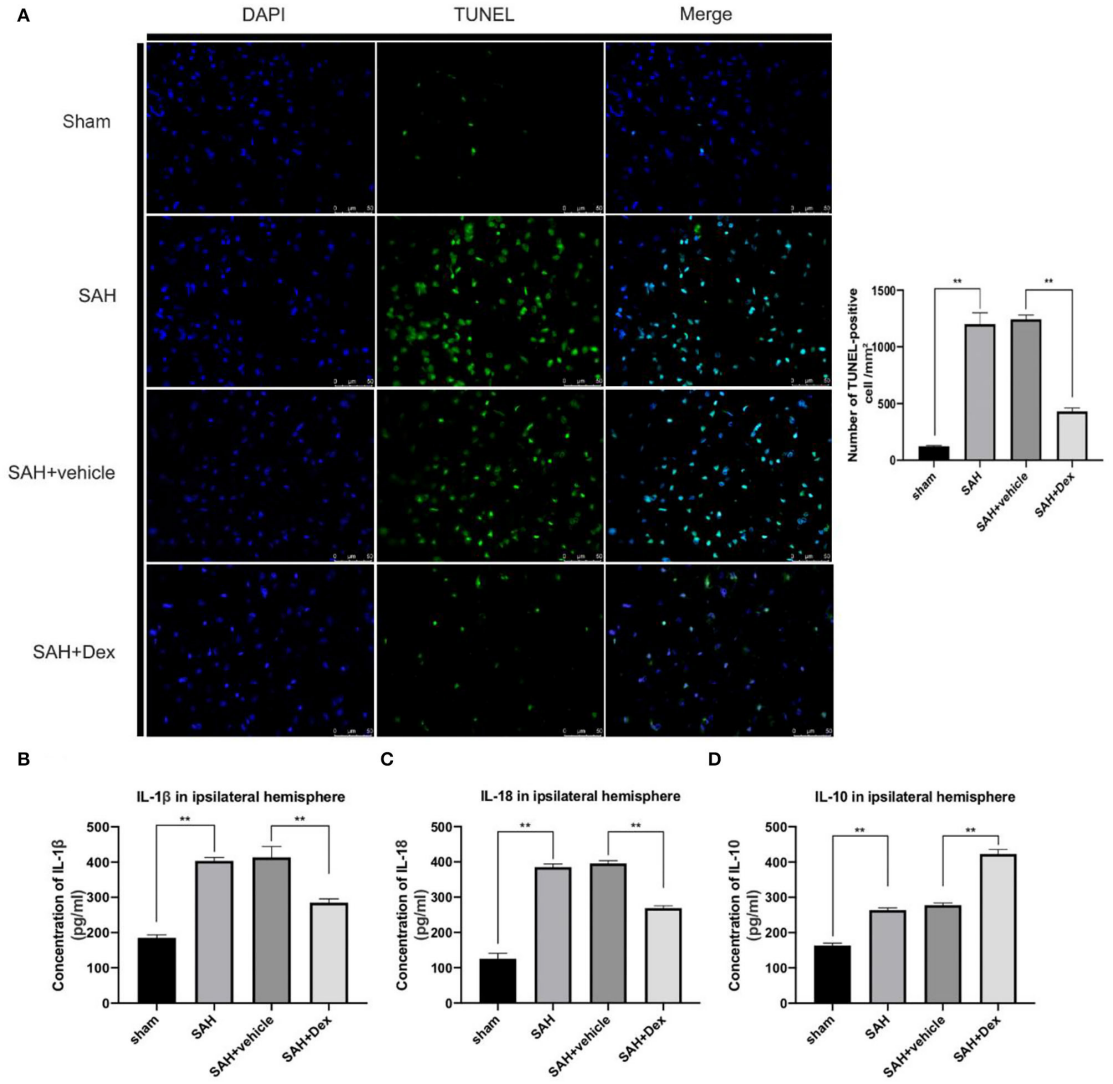
Effects of Dex on cell apoptosis and inflammatory factors production. **(A)** Representative microphotographs of TUNEL-positive cells in the ipsilateral cortex at 24 h after SAH. **(B–D)** ELISA results indicating the levels of the proinflammatory factors IL-1β, and IL-18 and the anti-inflammatory factor IL-10 in the ipsilateral cerebral tissue at 24 h post-SAH; *n* = 6 in each group. Data are expressed as the mean ± SD. **p < 0.01. Scale bar = 50 μm.

To further explore whether Dex could reduce apoptosis after SAH, we used the TUNEL technique. The results showed that the number of apoptotic cells was upregulated at 24 h after SAH compared with the sham group. Dex treatment significantly decreased the number of apoptotic cells compared with the SAH + vehicle group (Figure 7A). We next examined the effect of Dex treatment on the proinflammatory cytokines IL-1β and IL-18 and the anti-inflammatory cytokine IL-10. The results showed that SAH-induced upregulation of IL-1β and IL-18 was reduced (Figures 7B,C), whereas IL-10 was increased after Dex treatment (Figure 7D).

### The Positive Effect of Dex on the Reduction of GSDMD-Induced Pyroptosis Was Partially Inhibited by LY294002

**Figure 8:**
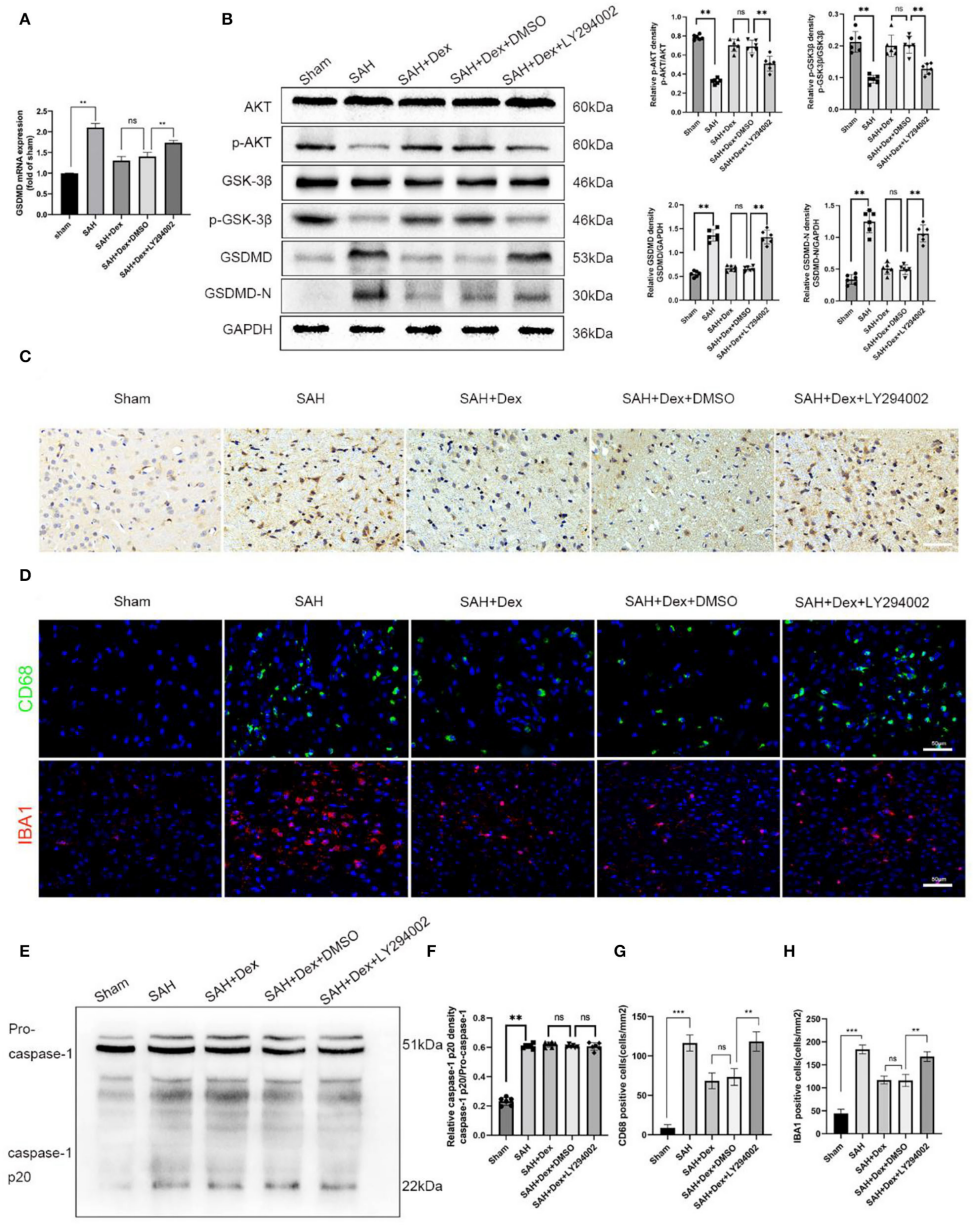
LY294002 inhibited Dex-induced upregulation of the PI3K/AKT/GSK3β pathway-related molecules, p-AKT, p-GSK3β at 24 h post-SAH and abolished the positive effect of Dex on the reduction of GSDMD and GSDMD-N. **(A)** Real-time RT-qPCR assay of GSDMD, *n* = 6 in each group. **(B)** Representative western blot images and quantitative analysis of western blot; *n* = 6 in each group; data are expressed as the mean ± SD. **p < 0.01. **(C)** Representative images of immunofluorescence staining of GSDMD in ipsilateral cerebral cortex. Scale bar = 50 μm. **(D,G,H)** Representative images of immunofluorescence staining and quantification of CD68 and IBA1 activation in ipsilateral cerebral cortex at 24 h post-SAH; Scale bar = 50 μm. *n* = 6 in each group; data are expressed as the mean ± SD. **p < 0.01. **(E,F)** Representative image of Western blot and quantification of caspase-1 p20 protein levels. **p < 0.01; ***p < 0.001.

Here, preliminary conclusions were obtained that Dex could effectively reduce GSDMD-induced pyroptosis in EBI after SAH and that the protective effects of Dex might involve the PI3K/AKT/GSK3β pathway. To confirm that Dex reduced GSDMD-induced pyroptosis *via* activating the PI3K/AKT/GSK3β pathway, we used the PI3K inhibitor LY294002. WB analyses revealed that LY294002 reduced the protein expression levels of p-AKT and p-GSK3β (Figure 8B). We found that co-administration of Dex and LY294002 enhanced the mRNA level of GSDMD (Figure 8A) and protein expression levels of GSDMD and GSDMD-N (Figure 8B) compared with the Dex + DMSO group at 24 h after SAH. Furthermore, IHC staining revealed that GSDMD was markedly increased at 24 h after SAH and that this increase was abrogated after administration of Dex. However, co-administration of LY294002 reversed Dex-mediated reductions in GSDMD (Figures 8B,C). We next examined the effect of Dex on activated microglia. As shown in (Figures 8D,G,H), LY294002 negated the suppression of Iba-1 and CD68-positive cells following DEX treatment. Importantly, the protein levels of caspase-1 p20 showed no significant difference between the SAH + Dex + DMSO and SAH + Dex + LY294002 groups (Figures 8E,F).

*In vitro* experiments were performed to further confirm whether LY294002 could abolish the positive effect on microglia pyroptosis by Dex. The increasements of p-AKT and p-GSK3β after Dex administration were all repressed by LY294002 in cultured microglia. In addition, the pyroptosis executioner GSDMD and GSDMD-N were elevated by LY294002 (Supplementary Figure 2A). The cultured microglia death was evaluated by double fluorescent staining with calcein AM and PI. Microglia treated with Dex and LY294002 demonstrated a significant increase in PI-positive cells in comparison with that in the Dex treatment group (Supplementary Figure 2B).

### Dex-Mediated Improvements in Brain Edema and Behavioral Deficits and Reductions in Neuronal Damage and Inflammatory Cytokine Expression Were Fully Abolished by LY294002

**Figure 9:**
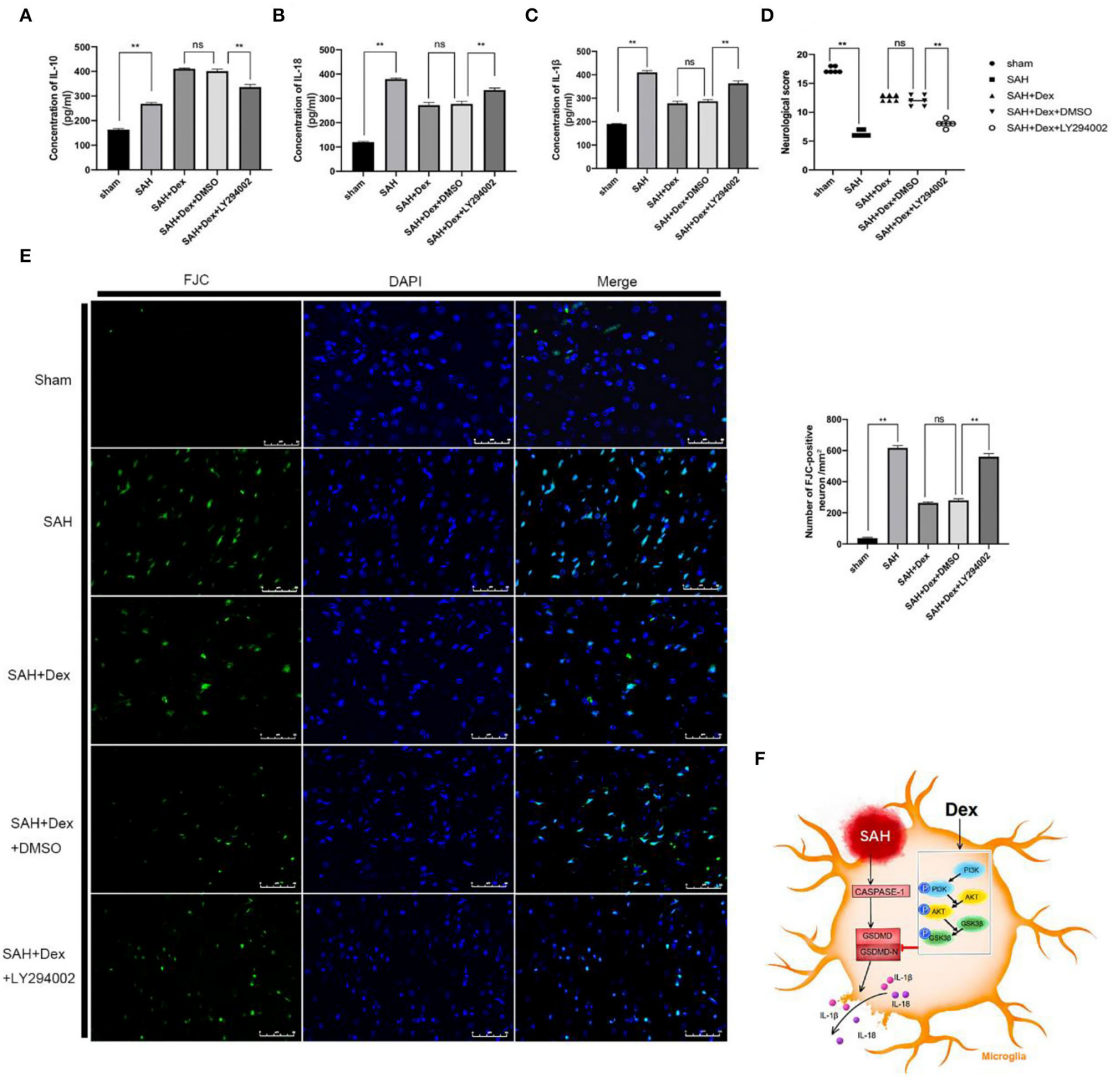
LY294002 abolished Dex effects on inflammatory cytokines production, neurobehavioral deficits, and neuronal injury after 24 h SAH. **(A–C)** ELISA results indicating the levels of the pro-inflammatory factors IL-1β, and IL-18 and the anti-inflammatory factor IL-10 in the ipsilateral cerebral tissue; *n* = 6 in each group. **(D)** Modified Garcia scores for each group; n = 6 in each group. **(E)** Representative FJC and DAPI staining images and quantitative analysis of FJC-positive cells in the ipsilateral cerebral cortex; *n* = 6 in each group. Data are expressed as the mean ± SD. **P < 0.01. Scale bar = 50 μm. **(F)** Graphical abstract of how Dex alleviates GSDMD-induced microglia pyroptosis after SAH *via* PI3K/AKT/GSK3β pathway.

We further evaluated the effects of LY294002 on behavioral deficits, neuronal damage, and inflammatory cytokine expression after Dex treatment at 24 h after SAH. LY294002 treatment abolished the effects of Dex-mediated upregulation of the anti-inflammatory factor IL-10 (Figure 9A) and inhibition of proinflammatory cytokines IL-18 (Figure 9B) and IL-1β (Figure 9C) at 24 h after SAH. The attenuation of neurobehavioral deficits associated with Dex treatment was also decreased following treatment of LY294002 (Figure 9D). Finally, the numbers of FJC-positive neurons were increased in the ipsilateral hemisphere of the Dex + LY294002 group compared with the SAH + Dex + DMSO group (Figure 9E).

## Discussion

In this study, we revealed that the beneficial effects of Dex against GSDMD-induced pyroptosis in EBI after SAH may be attributed to PI3K/AKT/GSK3β pathway activation. In the first experiment, our data demonstrated that GSDMD-induced pyroptosis reached a peak at 24 h after SAH. To further determine the mechanism of Dex on GSDMD-induced pyroptosis following SAH, we used rats as our experimental model. In our second experiment, we found that Dex administration at the doses of 25 μg/kg attenuated neurological deficits and brain water content and reduced the protein expression levels of GSDMD and GSDMD-N at 24 h after SAH. We thus chose a dose of 25 μg/kg for subsequent experiments. In our third experiment, the results showed that Dex administration, at 25 μg/kg, reduced FJC-positive neurons, BBB disruption, inflammatory cytokine expression, and the protein expression levels of GSDMD and GSDMD-N. Importantly, Dex activated the PI3K/AKT/GSK3β pathway at 24 h after SAH. In our fourth experiment, we found that the PI3K inhibitor LY294002 notably attenuated the positive effect of Dex at 24 h after SAH. Taken together, we hypothesized that Dex alleviates GSDMD-induced pyroptosis in EBI after SAH by reducing GSDMD and GSDMD-N and that this process involved activation of the PI3K/AKT/GSK3β pathway (Figure 9F).

Pyroptosis, a GSDMD-induced programmed cell death, is stimulated by a broad range of pathogens. GSDMD can be cleaved to generate both GSDMD-N and GSDMD-C by caspase-1 p20. GSDMD-N binds to the inner cell membrane and induces pore formation, which results in the release of inflammatory cytokines and pyroptosis-induced lytic cell death (Sborgi et al., 2016); therefore, GSDMD and GSDMD-N are the direct molecular regulators of pyroptosis. Indeed, in GSDMD-deficient cells, pyroptosis cannot be triggered (Shi et al., 2015). Recently, it has been proposed that pyroptosis should be redefined as GSDMD-mediated programmed necrosis, rather than as caspase-1-mediated necrosis (Shi et al., 2017). Most of the evidence has demonstrated that GSDMD plays a pivotal role in central nervous system injury (Liu et al., 2018; Zhang et al., 2019). In our study, we observed that GSDMD, GSDMD-N, and caspase-1 p20 were upregulated and reached a peak at 24 h after SAH, indicating that pyroptosis occurred after SAH, similar to the previous results (Yuan et al., 2020). Our results were in correspond to Xu et al. (2020) in which the authors found that pyroptosis occurred in microglia in the experimental SAH rat model. Thus, we assumed that GSDMD expression profiles in this study are mainly reflected by microglial pyroptosis. Our results indicated that the level of GSDMD and GSDMD-N was closely associated with the severity of brain injury after SAH. This result was in line with several studies, indicating that of GSDMD and GSDMD-N is reflective of disease progression and may thus be a therapeutic target (Tang et al., 2018; Wang et al., 2020).

Dexmedetomidine, a highly selective α2 adrenergic receptor agonist, is widely used in the intensive care unit. Recently, Okazaki et al. (2018) found that a low dosage of Dex during the first 24 h after admission was associated with positive neurological outcomes in patients with SAH. In that previous study, Dex was administered by continuous infusion titrated between 0.20 and 0.70 μg/kg/h by the bedside physician according to the patient heart rate and blood pressure. However, it is difficult to simulate this type of injection in rats. Several studies have reported the beneficial effects of Dex on various diseases with mechanisms of action attributed to anti-autophagy and inflammation regulation (Oh et al., 2019; Sun et al., 2019). Consistent with these previous studies, our experimental results showed that Dex decreased the expression of GSDMD and GSDMD-N and ameliorated brain edema, neurobehavioral deficits, and neuronal injury in a rat model of SAH. Recently, it is believed that the excessive neural cell apoptosis is caused by the reduction of elevated intracranial pressure, microcirculatory disturbance, ischemia–reperfusion, inflammatory injury, and oxidative stress in the EBI period after SAH (Serrone et al., 2015). We found that Dex significantly decreased the number of apoptotic cells, and this effect may be due to Dex reduced proinflammatory cytokines IL-1β and IL-18 which can cause inflammatory injury after SAH. Furthermore, a previous study showed that GSDMD is essential for the release of the proinflammatory cytokines IL-1β and IL-18 (He et al., 2015). Our data also showed that Dex significantly decreased the expression of IL-1β and IL-18 at 24 h after SAH. Furthermore, we investigated the underlying molecular mechanisms of the reduction of GSDMD after SAH by Dex.

It has been shown that the PI3K/AKT/GSK3β signaling pathway plays a pivotal role in alleviating early brain injury in stroke, including SAH and cerebral ischemia (Chen S. et al., 2014; Ma et al., 2016). A previous study has reported that Dex was able to alleviate hepatic ischemia–reperfusion injury *via* the PI3K/AKT/Nrf2-NLRP3 pathway (Wu et al., 2021). In this study, we also observed that Dex activated the PI3K /AKT/GSK3β pathway at 24 h after SAH. In the SAH group, and consistent with a previous study, we observed p-AKT and p-GSK3β-positive microglia by IF staining (Endo et al., 2006). LY294002, a PI3K inhibitor, has been widely used in many experiments to verify the mechanistic mediators of the PI3K/AKT/GSK3β pathway. Here, we used LY294002 to successfully inhibit Dex-induced activation of the PI3K/AKT/GSK3β and found it abolished the positive effects on pyroptosis executioner GSDMD associated with Dex after SAH. To exclude the other cells affected by Dex, we demonstrated PI3K/AKT/GSK3β pathway in cultured microglia. Xu et al. (2019) found that the caspase-1 inhibitor VX765 suppressed the expression of GSDMD. In another study, docosahexaenoic acid was found to activate the PI3K/AKT pathway and decrease the expression of caspase-1 p20 in hepatic ischemia–reperfusion injury, actions that were abolished by LY294002 (Li Z. et al., 2018). Interestingly, we found that the Dex-mediated activation of the PI3K/AKT/GSK3β pathway did not involve caspase-1 activity in our model of SAH. Thus, these results suggested that Dex reduced GSDMD-induced pyroptosis by activating the PI3K/AKT/GSK3β pathway, independent of caspase-1 activity.

There are several points in our study that need to be discussed. First, although the expression of caspase-1 p20, GSDMD, and GSDMD-N reached a peak at 24 h after SAH, their expression levels were still high at later time points. Our study only investigated the anti-pyroptosis effects of Dex and its associated mechanism of action in EBI after SAH. Second, due to the limitations of current technology, we could only determine the effect of Dex on pyroptosis by detecting the pyroptosis executioner GSDMD, as in previous studies. Finally, other mechanisms that Dex may be involved in the regulation of pyroptosis in SAH need to be further explored.

In conclusion, our data provided new evidence that pyroptosis of microglia is a crucial player in the pathophysiology of SAH. In addition, we demonstrated a novel mechanism by which Dex reduces pyroptosis executioner GSDMD after SAH. First, Dex alleviated GSDMD-induced pyroptosis of microglia in EBI after SAH, by decreasing the pyroptosis executioner GSDMD. Second, Dex reduced GSDMD by activating the PI3K/AKT/GSK3β pathway. Moreover, Dex ameliorated neurobehavioral deficits and neuronal injury in EBI after SAH. Taken together, our results suggest that Dex could represent a promising treatment against pyroptosis after SAH.

## Data Availability Statement

The raw data supporting the conclusions of this article will be made available by the authors, without undue reservation.

## Ethics Statement

The animal study was reviewed and approved by Southern Medical University Ethics Committee.

## Author Contributions

BW and LJ designed the study. BW, WL, LJ, and SG completed the experiments. HF, SG, and FJ performed the statistical analysis. BW and WL finished writing the manuscript. DF and CW revised the manuscript. CD and XL finished the revision. XZ and SS participated in discussion development and provided expert guidance. All authors contributed to the article and approved the submitted version.

## Funding

This work was supported by the National Natural Science Foundation Project (Grant Numbers: 81974178, 81974177, and 82001300).

## Conflict of Interest

The authors declare that the research was conducted in the absence of any commercial or financial relationships that could be construed as a potential conflict of interest.

## Publisher's Note

All claims expressed in this article are solely those of the authors and do not necessarily represent those of their affiliated organizations, or those of the publisher, the editors and the reviewers. Any product that may be evaluated in this article, or claim that may be made by its manufacturer, is not guaranteed or endorsed by the publisher.

## Supplementary Material

Supplementary Figure 1 Dex activated the PI3K/AKT/GSK3β pathway and mitigated pyroptosis in cultured BV-2 microglia. **(A)** Representative image of Western blot. **(B)** Representative images of co-immunofluorescence staining of GSDMD (green) and microglia (IBA1, red), Scale bar = 50 μm. **(C,D)** Quantitative analysis of western blot and immunofluorescence staining. n = 6 in each group. Data are expressed as mean ± SD. ^**^*p* < 0.01, ^***^*p* < 0.001.

Supplementary Figure 2 LY294002 abolished the positive effects of Dex in cultured BV-2 microglia. **(A)** Representative image of western blot. **(B)** Representative images of PI and calcein AM staining of microglia. Scale bar = 100 μm. **(C,D)** Quantitative analysis of western blot and immunofluorescence staining. n = 6 in each group. Data are expressed as mean ± SD. ^**^*p* < 0.01, ^***^*p* < 0.001.

Supplementary Table 1 The total number and mortality of rats in each group.

Supplementary Table 2 Modified Garcia scoring system.

Supplementary Table 3 The GSDMD primer sequences.

## Abbreviations

EBI: early brain injury
GSDMD: gasdermin D
Dex: dexmedetomidine
PI3K: phosphatidylinositol 3-kinase
AKT: protein kinase 3
GSK3β: glycogen synthase kinase-3
ANOVA: analysis of variance
BBB: blood–brain barrier
BWC: brain water content
CNS: central nervous system
DMSO: dimethylsulfoxide
DW: dry weight
EBI: early brain injury
ECA: external carotid artery
ELISA: enzyme-linked immunosorbent assay
FJC: Fluoro-Jade C
H&E: hematoxylin and eosin
Iba1: ionized calcium-binding adapter molecule1
ICA: internal carotid artery
IF: immunofluorescence
IHC: immunohistochemistry
IL: interleukin
PBS: phosphate buffer solution
qRT-PCR: quantitative real-time polymerase chain reaction
RT: room temperature
SAH: subarachnoid hemorrhage
WW: wet weight
TEM: transmission electron microscopy.
