# Supplementary figures and images for: Dexmedetomidine Inhibits Gasdermin D-Induced Pyroptosis via the PI3K/AKT/GSK3β Pathway to Attenuate Neuroinflammation in Early Brain Injury After Subarachnoid Hemorrhage in Rats
Source: Front Cell Neurosci. 2022 Jun 21;16:899484. doi: 10.3389/fncel.2022.899484 (PMC9253293; doi:10.3389/fncel.2022.899484)

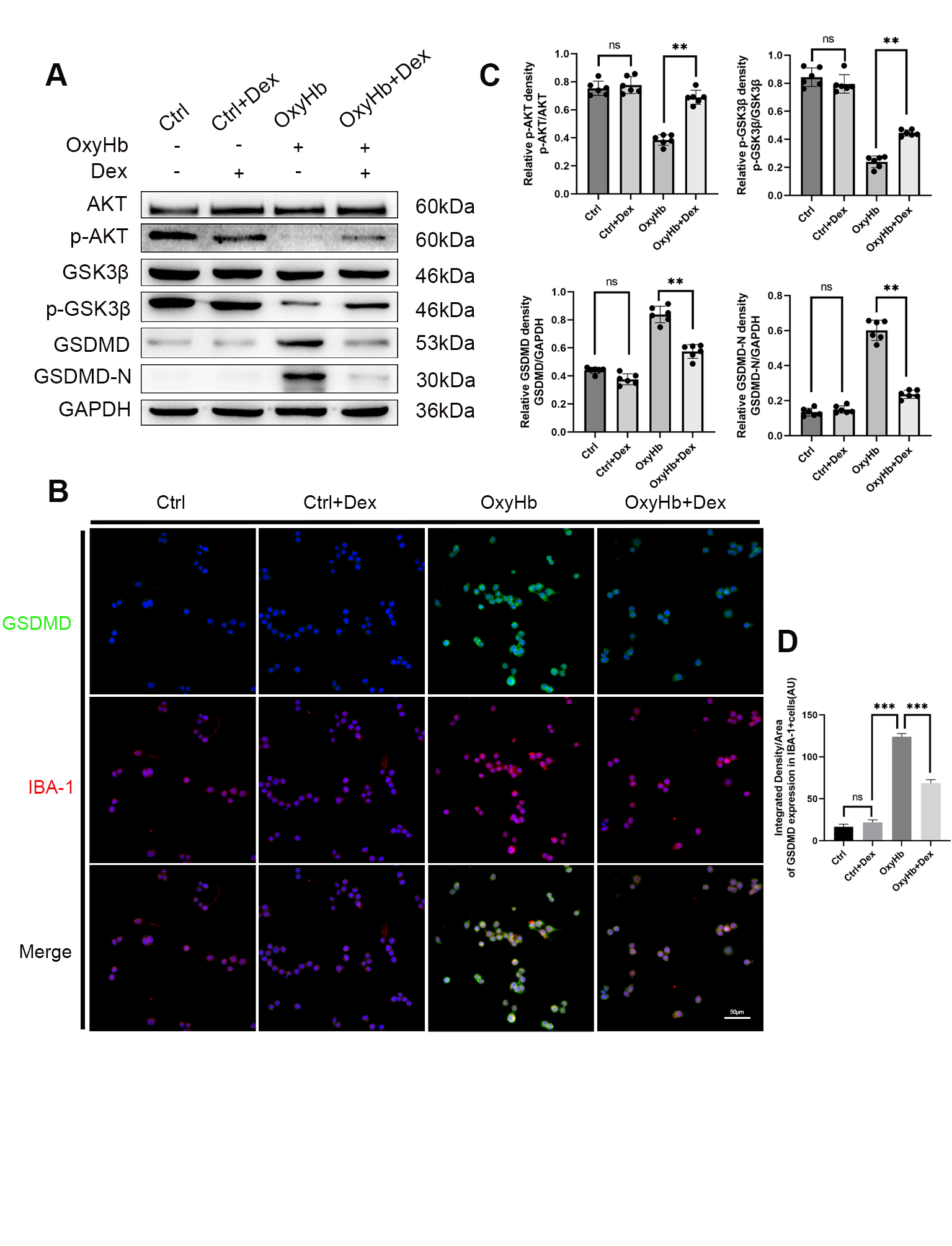

Supplement: Supplementary Figure 1 — Dex activated the PI3K/AKT/GSK3β pathway and mitigated pyroptosis in cultured BV-2 microglia. (A) Representative image of Western blot. (B) Representative images of co-immunofluorescence staining of GSDMD (green) and microglia (IBA1, red), Scale bar = 50 μm. (C,D) Quantitative analysis of western blot and immunofluorescence staining. n = 6 in each group. Data are expressed as mean ± SD. **p < 0.01, ***p < 0.001. [file Image_1.TIF]

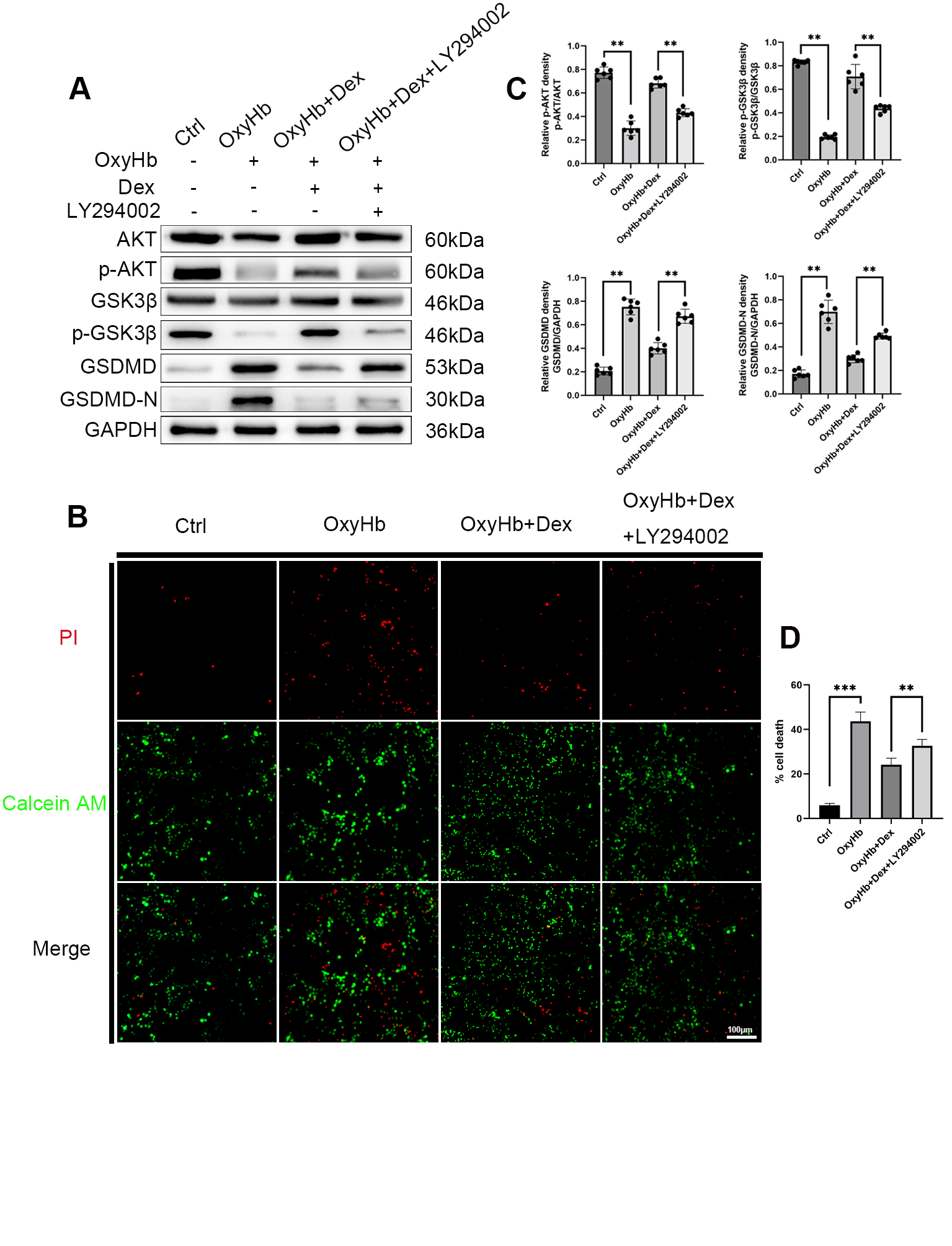

Supplement: Supplementary Figure 2 — LY294002 abolished the positive effects of Dex in cultured BV-2 microglia. (A) Representative image of western blot. (B) Representative images of PI and calcein AM staining of microglia. Scale bar = 100 μm. (C,D) Quantitative analysis of western blot and immunofluorescence staining. n = 6 in each group. Data are expressed as mean ± SD. **p < 0.01, ***p < 0.001. [file Image_2.TIF]
