# Supplementary material for: Dexmedetomidine Inhibits Gasdermin D-Induced Pyroptosis via the PI3K/AKT/GSK3β Pathway to Attenuate Neuroinflammation in Early Brain Injury After Subarachnoid Hemorrhage in Rats
Source: Front Cell Neurosci. 2022 Jun 21;16:899484. doi: 10.3389/fncel.2022.899484 (PMC9253293; doi:10.3389/fncel.2022.899484)
Supplement: Supplementary Table 1 — The total number and mortality of rats in each group. [file Table_1.DOCX]

**Supplementary**

**Table.1**

|  | Total number of rats | Number of deaths | Number of SAH grade<8 | Total number of rats exclusion |
| --- | --- | --- | --- | --- |
| sham | 88 | 0 | - | 0 |
| SAH | 172 | 36 | 26 | 62 |
| SAH + vehicle-1 | 44 | 9 | 8 | 17 |
| SAH+DEX (15μg/kg) | 14 | 3 | 2 | 5 |
| SAH+DEX (25μg/kg) | 74 | 17 | 13 | 30 |
| SAH+DEX (45μg/kg) | 14 | 4 | 2 | 6 |
| SAH+DEX (25μg/kg) + vehicle-2 | 30 | 7 | 7 | 14 |
| SAH + DEX (25μg/kg) + LY294002 | 30 | 8 | 8 | 16 |
