# Supplementary material for: Dexmedetomidine Inhibits Gasdermin D-Induced Pyroptosis via the PI3K/AKT/GSK3β Pathway to Attenuate Neuroinflammation in Early Brain Injury After Subarachnoid Hemorrhage in Rats
Source: Front Cell Neurosci. 2022 Jun 21;16:899484. doi: 10.3389/fncel.2022.899484 (PMC9253293; doi:10.3389/fncel.2022.899484)
Supplement: Supplementary Table 2 — Modified Garcia scoring system. [file Table_2.DOCX]

**Table.2**

| Test | score |  |  |  |
| --- | --- | --- | --- | --- |
|  | 0 | 1 | 2 | 3 |
| Spontaneous activity (in cage for 5 min) | No movement | Barely moves | Moves but does not approach at least three sides of cage | Moves and approaches at least three sides of cage |
| Symmetry of movements (four limbs) | Left side: no movement | Left side: slight movement | Left side: moves slowly | Both sides: move symmetrically |
| Symmetry of forelimbs (outstanding while held by tail) | Left side: no movement, no outreaching | Left side: slight movement to outreach | Left side: moves and outreaches less than right side | Symmetrical outreach |
| Climbing wall of wire cage | _ | Fails to climb | Left side is weak | Normal climbing |
| Reaction to touch on either side of trunk | _ | No response on left side | Weak response on left side | Symmetrical response |
| Response to vibrissae touch | _ | No response on left side | Weak response on left side | Symmetrical response |
