# Supplementary material for: Dexmedetomidine Inhibits Gasdermin D-Induced Pyroptosis via the PI3K/AKT/GSK3β Pathway to Attenuate Neuroinflammation in Early Brain Injury After Subarachnoid Hemorrhage in Rats
Source: Front Cell Neurosci. 2022 Jun 21;16:899484. doi: 10.3389/fncel.2022.899484 (PMC9253293; doi:10.3389/fncel.2022.899484)
Supplement: Supplementary Table 3 — The GSDMD primer sequences. [file Table_3.DOCX]

**Supplementary**

**Table.3**

Real-time PCR primers used for quantification of mRNA expression in this study

| Primer name Sequence | (5′ → 3′) |  |
| --- | --- | --- |

| GSDMD | Forward | AGATCGTGGATCATGCCGTC |
| --- | --- | --- |
|  | Reverse | CCCGATGGAATGGAGTACGG |
